# Improved coverage and timing of childhood vaccinations in two post-Soviet countries, Armenia and Kyrgyzstan

**DOI:** 10.1186/s12889-015-2091-9

**Published:** 2015-08-19

**Authors:** A. Schweitzer, G. Krause, F. Pessler, M. K. Akmatov

**Affiliations:** Helmholtz Centre for Infection Research, Braunschweig, Germany; Hannover Medical School, Hannover, Germany; TWINCORE Centre for Experimental and Clinical Infection Research, Hannover, Germany

**Keywords:** Vaccination coverage, Timing of vaccination, Demographic and Health Survey, Post-Soviet countries, Armenia, Kyrgyzstan

## Abstract

**Background:**

Timing of childhood vaccinations has received close attention in many countries. Little is known about the trends in correctly timed vaccination in former Soviet countries. We examined trends in vaccination coverage and correct timing of vaccination in two post-Soviet countries, Armenia and Kyrgyzstan, and analyzed factors associated with delayed vaccinations.

**Methods:**

We used data from the Demographic and Health Surveys; the surveys were conducted in 2000 (*n =* 1726), 2005 (*n =* 1430) and 2010 (*n =* 1473) in Armenia and in 1997 (*n =* 1127) and 2012 (*n =* 4363) in Kyrgyzstan. We applied the Kaplan-Meier method to estimate age-specific vaccination coverage with diphtheria, tetanus and pertussis (DTP) vaccine and a measles-containing vaccine (MCV). A Cox proportional hazard regression with shared frailty was used to examine factors associated with delayed vaccinations.

**Results:**

Vaccination coverage for all three doses of the DTP vaccine increased in Armenia from 92 % in 2000 to 96 % in 2010. In Kyrgyzstan, DTP coverage was 96 % and 97 % in 1997 and 2012, respectively. Vaccination coverage for MCV increased from 89 % (Armenia, 2000) and 93 % (Kyrgyzstan, 1997) to 97 % (Armenia, 2010) and 98 % (Kyrgyzstan, 2012). The proportion of children with correctly timed vaccinations increased over time for all examined vaccinations in both countries. For example, the proportion of children in Armenia with correctly timed first DTP dose (DTP1) increased from 46 % (2000) to 66 % (2010). In Kyrgyzstan, the proportion of correctly timed DTP1 increased from 75 % (1997) to 87 % (2012). In Armenia, delays in the third DTP dose (DTP3) and MCV vaccinations were less likely to occur in the capital, whereas in Kyrgyzstan DTP3 and MCV start was delayed in the capital compared to other regions of the country. Also, in Armenia living in urban areas was associated with delayed vaccinations.

**Conclusions:**

Vaccination coverage and timing of vaccination improved over the last years in both countries. Further efforts are needed to reduce regional differences in timely vaccinations.

## Background

Achieving high vaccination coverage is a necessary, but an insufficient indicator of the quality of vaccination programs geared towards preventing childhood infectious diseases. The timing of vaccination is increasingly recognized as another important target for optimal protection of children [1] and has received close attention in many countries in recent years [2, 3]. The standard measure of vaccination coverage and also compliance with recommended vaccinations is usually estimated based on the percentage of children in a specific age group who have received the recommended number of vaccine doses without regard to the timing of vaccination [4, 5]. Studies have demonstrated that high vaccination coverage rates do not necessarily imply correctly timed vaccinations [3, 6–9]. According to Clark et al., timing of childhood vaccinations varies widely among and within countries, and published yearly estimates of national coverage do not capture these variations [3]. Incorrectly timed (early or delayed) vaccination doses might in fact explain the persistence or even the resurgence of vaccine-preventable infections, which is especially relevant for countries where high levels of vaccination coverage at milestone ages have been achieved [10, 11]. Outbreaks of diseases such as measles can occur in a population with high vaccination coverage [12]. This has been attributed to vaccine failure resulting from individuals being vaccinated outside the recommended period [13].

The timely start of vaccination is important in light of the rapid waning of transplacental immunity in the first year of life against vaccine-preventable diseases such as pertussis and invasive *Haemophilus influenzae* type b disease [8]. Delayed doses in turn put individuals at risk of disease due to waning immunity over time and put the whole community at risk of epidemics [14]. Thus, it is important to take the correct timing of vaccination into account, as relying exclusively on vaccination coverage alone can lead to a false assumption of disease protection [6, 10].

In a previous analysis of childhood vaccination in several post-Soviet countries, we observed that a substantial proportion of children was vaccinated with delays [2, 15]. Little is known about the trends in vaccination coverage and correctly timed vaccination over time in these countries. This is especially important since health care systems in these countries underwent a deep transition. After the collapse of the Soviet Union, the health care systems of the Newly Independent States (NIS) moved from the *Semashko* model, a centralized health-care system, to a range of institutional, financial arrangements and out-of-pocket payments [16]. These factors, in turn, may contribute to a decrease in health-care utilization, particularly among the poor, which, together with the level of poverty and decaying socio-economic health infrastructure, alter the morbidity profile [16]. These changes were reflected in sharp drops in vaccination coverage in the early 1990s in the Central Asian and Caucasian republics [17, 18]. Increased outbreaks of some vaccine-preventable diseases, such as diphtheria and measles, were observed in the mid 1990s although vaccination coverage increased to up to 90 % in the NIS after 1995 [17, 19], which could be accounted for by delayed vaccinations.

In the current analysis we used data from two post-Soviet countries (Armenia and Kyrgyzstan) that allow tracing changes in vaccination coverage and timing of vaccination over time. Both countries have adopted the WHO guidelines for childhood vaccinations [20, 21]. These guidelines call for all children to receive the following: a BCG vaccine against tuberculosis; three doses of the diphtheria, pertussis, and tetanus (DPT) vaccine, three doses of polio vaccine, and a measles vaccine during the first year of life [22]. Specifically, we aimed to estimate the trends in vaccination coverage and correctly timed vaccination for selected childhood vaccinations over time and to analyze factors associated with delayed vaccinations.

## Methods

We used data from the Demographic and Health Surveys (DHS) in two countries, Armenia and Kyrgyzstan, where multiple surveys over a period of several years were available. The surveys were conducted in 2000, 2005 and 2010 in Armenia and in 1997 and 2012 in Kyrgyzstan. DHS are nationally representative household surveys that provide data for a wide range of monitoring and impact evaluation indicators in the areas of population, health, and nutrition [14]. These surveys provide the most recent information about vaccination coverage in some post-Soviet countries and are independent of the official health reports, which are known to overestimate vaccination coverage [18].

A multi-stage sampling technique was used in the DHS surveys to obtain representative samples in both countries. In the first stage, sampling areas were selected separately in urban and rural areas. In rural areas a village was the sampling unit. Urban areas are subdivided into “health blocks”, i.e. districts for which doctors from local clinics are responsible. A list of all households was obtained from the respective authorities. In the second stage, households with women of reproductive age between 15 and 49 years were randomly selected. Information about reproductive health of women, infant and child mortality, nutrition of women and children, and vaccination data were collected in the surveys using standard DHS questionnaires [14].

Vaccination data were obtained mostly from child health cards available at local health care facilities or by information recalled by the mother in the event that the mother did not have a child health card or an immunization was not recorded on the card. We used data on three doses of diphtheria, tetanus and pertussis (i.e. DTP1, DTP2 and DTP3) vaccine and the first dose of a measles-containing vaccine (MCV). The Polio vaccine was omitted from the analysis because it is given according to the same schedule as DTP; consequently, the timing of administration is expected to be the same for both, and most children either received both or neither.

We assessed vaccination coverage and timing of vaccination in accordance with the respective national immunization schedules of Armenia and Kyrgyzstan. We defined vaccinations as correctly timed if administered within 4 weeks after the recommended age specified in the national immunization schedule (Table 1). We defined up-to-date (UTD) vaccination coverage as the proportion of children vaccinated between 12 and 59 months of age for DTP vaccines and between 18 and 59 months of age for MCV.

**Table 1:** Recommended age for DTP and MCV vaccinations for children in Armenia and Kyrgyzstan as per the WHO guidelines for childhood vaccinations

	Vaccines			
Country	DTP1	DTP2	DTP3	MCV
Armenia	3 months	4.5 months	6 months	12 months
Kyrgyzstan	2 months	3.5 months	5 months	12 months

### Statistical analysis

Initially, we assessed UTD vaccination coverage using data procured from health cards and/or recalled by the parents. This analysis included a sample of children between 12 and 59 months of age for DTP vaccines and between 18 and 59 months for MCV. Furthermore, we applied the Kaplan–Meier method to estimate vaccination coverage at any given age. For this analysis we did not restrict the sample, i.e. all children between 0 and 59 months were included. Data from all children for whom complete information on birthdate and dates of vaccination were available were used for this analysis. If vaccination had not been received by the day of interview, the case was considered as censored. The survival function S(age), i.e. the proportion of children not vaccinated at the end of an age interval divided by those not vaccinated at the beginning of the age interval, was estimated for each interval. At any given age, cumulative vaccination coverage was calculated as 1 − S(age). Finally, we applied a Cox proportional hazard regression with shared frailty to account for variation within clusters to examine factors associated with vaccination delays (two separate models for DTP3 and MCV vaccinations). A frailty is a latent random effect that enters multiplicatively on the hazard function [23]. The models were adjusted for the following variables: child’s gender, place of residence, birth order, mother’s age, education, household’s wealth index, and region. In addition, the models were adjusted for child’s year of birth to control for unmeasured birth cohort effects. The analysis was done with the statistical programs SPSS for Windows, version 19 (IBM Corporation, Armonk, NY, United States) and STATA for Windows, version 12 (StataCorp LP, Texas, United States).

### Ethical approval

The analysis of this study was based on existing survey data collected by the DHS (The DHS Programme, www.dhsprogram.com). All surveys included in the analysis were approved by the Institutional Review Board of ICF International in Calverton, MD, USA. Study participants provided informed consent before participation. Survey data were provided by ICF International, Inc.

## Results

### Characteristics of the samples

In both countries, the proportion of the study population born in a health care facility and with higher maternal education level increased from 2000 to 2010 (Armenia) and from 1997 to 2012 (Kyrgyzstan), respectively (Table 2). The proportion of available vaccination cards in Armenia remained between 91 and 93 % without a clear trend, while it increased in Kyrgyzstan from 76 % to 89 %.

**Table 2:** Selected socio-demographic characteristics of the samples, Demographic and Health Surveys in Armenia and Kyrgyzstan (%)

	Armenia 2000 *n = 1726*	Armenia 2005 *n = 1430*	Armenia 2010 *n = 1473*	Kyrgyzstan 1997 *n = 1127*	Kyrgyzstan 2012 *n = 4363*
Gender					
Male	56.4	53.6	52.6	51.1	51.4
Female	43.6	46.4	47.4	48.9	48.9
Childhood place of residence					
Urban	43.9	67.0	65.5	25.4	25.4
Rural	56.1	33.0	34.5	74.6	74.6
Child’s place of birth					
Delivery at home	8.5	1.9	0.2	3.2	0.4
Delivery in health care facility	91.5	98.1	99.8	96.8	99.6
Mother’s education					
Primary	0.2	0.3	5.4	0.2	0.3
Secondary	84.5	78.3	37.7	86.0	55.9
Higher	15.4	21.4	56.9	13.8	44.1
Vaccination cards available					
No	7.6	8.5	6.9	24.4	11.3
Yes	92.4	91.5	93.1	75.6	88.7

### Up-to-date (UTD) vaccination coverage

**Fig. 1:**
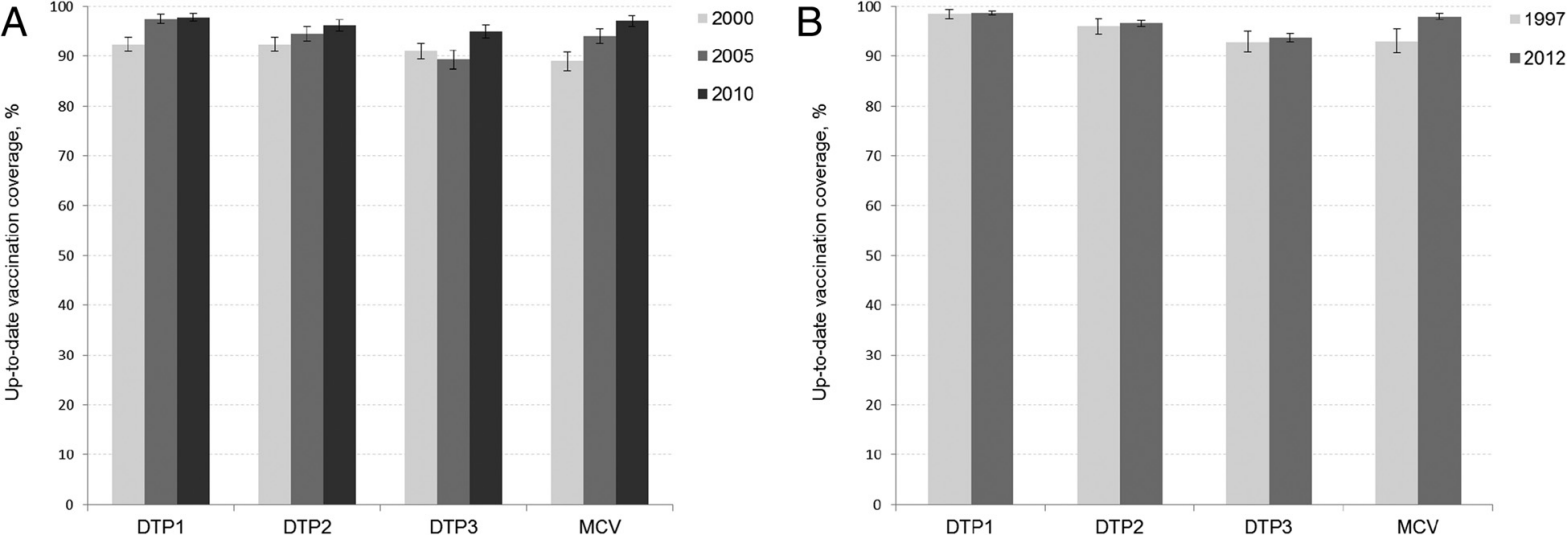
Changes in up-to-date vaccination coverage over survey years*. *Up-to-date vaccination coverage among children aged between 12 and 60 months for the DTP vaccines and between 18 and 60 months for a measles-containing vaccine. Information on coverage is based on immunization records and maternal reports

UTD vaccination coverage for all three doses of the DTP vaccine increased significantly in Armenia from 92 % in 2000 to 96 % in 2010 (Fig. 1; p for trend <0.0001 [DTP1], <0.0001 [DTP2], and 0.001 [DTP3]). In Kyrgyzstan, DTP coverage was 96 % and 97 % in 1997 and 2012, respectively (Fig. 1; p for trend 0.76 [DTP1], 0.52 [DTP2], and 0.41 [DTP3]). In both countries, the UTD vaccination coverage with MCV was less than 90 % in the older surveys and increased over the survey years, reaching the mark of 95 % (p for trend <0.0001 in both countries).

### Changes over time in correctly timed vaccination

**Fig. 2:**
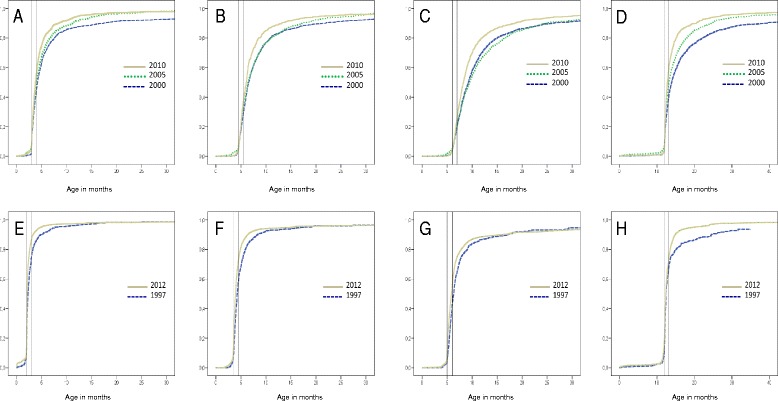
Cumulative vaccination coverage (inverse Kaplan–Meier estimates). **a**. DTP1; **b**. DTP2; **c**. DTP3 and **d**. MCV in Armenia. **e**. DTP1; **f**. DTP2, **g**. DTP3 and **h**. MCV in Kyrgyzstan. The vertical reference line indicates age at vaccination recommended by national vaccination schedules in the respective countries

In both countries, the proportion of correctly timed vaccinations increased considerably over time for all examined vaccinations (Table 3). The largest increase in correctly timed vaccinations was observed for MCV in Armenia, increasing from 39 % in the survey 2000 to 62 % in 2012, amounting to an increase of 59 %. In Armenia, 46 %, 51 % and 66 % of children received DTP1 before the age of 3 months in 2000, 2005 and 2012, respectively (Table 3, Fig. 2). A similar trend was observed in Kyrgyzstan: 75 % and 87 % of children received DTP1 before the age of 2 months in 1997 and 2012, respectively. The median time of delay (weeks) decreased in both countries (Table 3). The proportion of children with correctly timed vaccinations was higher in Kyrgyzstan than in Armenia (Fig. 2).

**Table 3:** Estimates of correctly timed vaccination according to the recommended ages at vaccination (%) and median delays (weeks)

	DTP1		DTP2		DTP3		MCV	
Armenia	Correctly timed vaccination, %^a^	Median delay in weeks	Correctly timed vaccination, %^a^	Median delay in weeks	Correctly timed vaccination, %^a^	Median delay in weeks	Correctly timed vaccination, %^a^	Median delay in weeks
Survey 2000	46	6.3	29	10.7	22	16.4	39	8.7
Survey 2005	51	5.3	33	10.0	22	17.4	48	5.7
Survey 2010	66	3.0	50	6.1	40	9.6	62	3.3
Kyrgyzstan								
Survey 1997	75	2.0	59	4.6	46	7.4	67	2.3
Survey 2012	87	1.1	73	3.0	59	5.6	73	1.9

^a^Vaccination was considered to be timed correctly if administered within 4 weeks after the recommended age specified in the national immunisation schedule

### Factors associated with time to start of vaccinations

In Armenia, living in urban areas was associated with delays with both DTP3 and MCV vaccinations (Table 4, second and third columns). Additionally, maternal age was associated with correctly timed DTP3 and MCV vaccinations, and children of younger mothers were more likely to have correctly timed vaccinations. Regional differences in timely vaccinations were found in both countries. In Armenia, DTP3 and MCV vaccinations were more likely to be delayed in the Armavir and Syunik regions than in the capital city, Yerevan. In Kyrgyzstan, the association was reversed, i.e. DTP3 vaccination was more likely to be delayed in Bishkek, the capital city, compared to other regions; MCV was delayed in the capital Bishkek compared to the regions of Issyk-Kul, Djalal-Abad, Talas and Osh.

**Table 4:** Factors associated with time to start vaccinations (hazard ratios and 95 % confidence intervals from multivariable Cox proportional hazard regression with shared frailty)^a^

	Armenia, 2010		Kyrgyzstan, 2012	
	DTP3	MCV	DTP3	MCV
Gender	*HR (95 % CI)*	*HR (95 % CI)*	*HR (95 % CI)*	*HR (95 % CI)*
Male vs. female	0.89 (0.78–1.02)	0.94 (0.82–1.08)	1.03 (0.95–1.10)	0.94 (0.87–1.02)
Childhood place of residence				
Urban vs. rural	0.73 (0.57–0.92)	0.83 (0.67–1.03)	1.01 (0.82–1.25)	1.03 (0.85–1.23)
Birth order				
1 vs. 3+	0.63 (0.40–1.01)	0.97 (0.60–1.55)	1.01 (0.87–1.17)	1.02 (0.87–1.20)
2 vs. 3+	0.61 (0.39–0.96)	0.84 (0.52–1.34)	0.96 (0.84–1.11)	1.00 (0.86–1.15)
3 vs. 3+	0.55 (0.34–0.89)	0.80 (0.50–1.29)	1.04 (0.92–1.18)	1.10 (0.96–1.26)
Mother’s age				
15–19 vs. 45–49	6.14 (1.76–21.42)	3.42 (0.89–13.17)	1.19 (0.69–2.04)	1.04 (0.51–2.09)
20–24 vs. 45–49	5.39 (1.73–16.83)	2.51 (0.80–7.82)	0.95 (0.65–1.39)	1.10 (0.75–1.61)
25–29 vs. 45–49	5.24 (1.70–16.19)	2.76 (0.90–8.54)	0.91 (0.63–1.31)	1.13 (0.78–1.62)
30–34 vs. 45–49	4.77 (1.54–14.73)	2.47 (0.80–7.64)	0.89 (0.62–1.28)	1.12 (0.78–1.60)
35–39 vs. 45–49	4.52 (1.44–14.17)	2.31 (0.74–7.23)	0.92 (0.64–1.33)	1.11 (0.77–1.59)
40–44 vs. 45–49	4.93 (1.46–16.75)	2.26 (0.67–7.60)	1.00 (0.68–1.47)	1.23 (0.84–1.79)
Mother’s education				
Secondary vs. higher	0.95 (0.82–1.10)	0.91 (0.78–1.07)	0.95 (0.88–1.03)	1.01 (0.92–1.10)
Wealth index				
Poorest vs. richest	0.92 (0.65–1.30)	0.97 (0.69–1.35)	1.14 (0.89–1.46)	0.89 (0.69–1.13)
Poor vs. richest	1.08 (0.81–1.45)	1.01 (0.76–1.34)	1.15 (0.90–1.47)	0.85 (0.66–1.09)
Middle vs. richest	0.96 (0.73–1.26)	0.88 (0.68–1.14)	1.15 (0.90–1.47)	0.91 (0.71–1.16)
Rich vs. richest	1.03 (0.80–1.33)	0.95 (0.74–1.21)	1.13 (0.92–1.40)	0.92 (0.75–1.14)
Region				
Aragatsotn vs. Yerevan (capital)	1.00 (0.67–1.49)	0.98 (0.69–1.40)	-	-
Ararat vs. Yerevan (capital)	0.84 (0.56–1.27)	0.85 (0.59–1.21)	-	-
Armavir vs. Yerevan (capital)	0.60 (0.40–0.91)	0.71 (0.49–1.02)	-	-
Gegharkunik vs. Yerevan (capital)	1.05 (0.72–1.54)	0.74 (0.53–1.04)	-	-
Lori vs. Yerevan (capital)	0.81 (0.52–1.24)	0.89 (0.62–1.29)	-	-
Kotayk vs. Yerevan (capital)	0.87 (0.60–1.28)	0.86 (0.62–1.20)	-	-
Shirak vs. Yerevan (capital)	1.31 (0.90–1.91)	1.23 (0.89–1.72)	-	-
Syunik vs. Yerevan (capital)	0.39 (0.26–0.60)	0.38 (0.26–0.55)	-	-
VayotsDzor vs. Yerevan (capital)	0.77 (0.51–1.16)	0.77 (0.53–1.10)	-	-
Tavush vs. Yerevan (capital)	0.86 (0.59–1.26)	0.72 (0.52–1.01)	-	-
Issyk-Kul vs. Bishkek (capital)	-	-	2.93 (2.10–4.10)	1.34 (1.01–1.79)
Djalal-Abad vs. Bishkek (capital)	-	-	4.03 (2.89–5.64)	1.76 (1.33–3.32)
Naryn vs. Bishkek (capital)	-	-	1.96 (1.38–2.77)	1.21 (0.90–1.62)
Batken vs. Bishkek (capital)	-	-	1.94 (1.39–2.71)	0.94 (0.70–1.25)
Osh Oblast vs. Bishkek (capital)	-	-	1.59 (1.13–2.23)	1.24 (0.93–1.65)
Talas vs. Bishkek (capital)	-	-	2.49 (1.78–3.50)	1.53 (1.15–2.04)
Chui vs. Bishkek (capital)	-	-	2.55 (1.79–3.62)	1.10 (0.82–1.48)
Osh City vs. Bishkek (capital)	-	-	2.73 (2.00–3.71)	1.57 (1.20–2.05)

*HR* hazard ratio, *CI* confidence intervals

^a^Adjusted for child’s year of birth and other variables listed in the table

## Discussion

To our knowledge, this is the first paper to investigate trends in vaccination coverage and correct timing of vaccinations in the former Soviet Republics of Armenia and Kyrgyzstan. Based on survey data from 2000, 2005 and 2010 in Armenia and from 1997 and 2012 in Kyrgyzstan, we observed that vaccination coverage and correct timing improved over time in both countries. The improvement was more prominent in Armenia when comparing earlier surveys in both countries. To start with, Kyrgyzstan had a better and relatively higher vaccination coverage and better timing than Armenia. A 59 % increase in correct vaccination timing in Armenia was reported in MCV over the last decade in Armenia. Furthermore, available incidence data on measles, diphtheria and tetanus from Armenia and Kyrgyzstan corroborate our findings. No cases of diphtheria have been reported in the past few years in both countries [24]. The incidence of measles has dropped substantially in Kyrgyzstan, from 21 per 100,000 in 1997 to 0 per 100,000 in 2012 [24]. Similarly, in Armenia, the incidence of measles has dropped from 71 per 100,000 in 2005 to 0 per 100,000 in 2012. The last measles outbreak reported in either country was in Kyrgyzstan in 2011. These changes could well be accounted for by the improvement in vaccination coverage and timing. Based on the aforementioned data and our analysis one could conclude that there has been a reduction in vaccine-preventable diseases in both countries.

This evolution could in part be accounted for by an increased focus and prioritization of the elimination of vaccine-preventable diseases in these two countries. The elimination of measles and rubella remains a public health priority in Armenia and Kyrgyzstan. The World Health Organization (WHO) together with other international organizations such as the United Nations Children’s Fund (UNICEF) implemented a global strategy for elimination of measles and rubella, and both countries are also a party to this strategy. In particular, elimination of measles and rubella is part of the public health strategy of Armenia [25]. For measles elimination, achieving 95 % vaccination coverage would be an important step [26]. According to Lernout et al., adherence to age recommendations is harder to achieve for DTP than for measles [1]. This can be due to the stricter vaccination schedule for this vaccine, i.e. frequency of doses, and is reflected in the lower proportions of correctly timed vaccinations reported for consecutive doses of DTP (dose 2 and 3) [1]. That said, in our study improved timing of consecutive DTP doses was observed over time in both countries. The results of our analysis are encouraging as they clearly suggest that both countries have achieved the recommended levels (95 % for MCV) of coverage for the elimination of infections such as measles. Although our results suggest that progress has been made, additional efforts are needed. We observed that the up-to-date coverage for DTP2 and DTP3 was still quite low in both countries. This has important public health implications since transmission is likely to continue to pose risks of outbreaks in unvaccinated or incompletely vaccinated communities. This was evident in the diphtheria outbreak reported in the 1990s in the republics of the former Soviet Union [17, 18]. Maintaining high vaccination coverage is hence essential to prevent outbreaks and sustain disease elimination.

In Armenia, outpatient care is provided by urban polyclinics, rural health centers/ambulatories, and *feldsher-accoucher* posts (physician’s assistant/midwife) [20]. In Kyrgyzstan, primary health care, which includes childhood vaccination, is provided through *feldsher-accoucher* posts, groups of family doctors, family medicine centers, and general practice centers [21]. These providers focus primarily on disease prevention, vaccination, antenatal care services, etc. [21]. Sufficient supplies of vaccine at the health clinics and appropriate health care provider reminder and recall systems are essential for efficient vaccine delivery [27].

Vaccination timing, meaning the receipt of all scheduled vaccinations in an age-appropriate fashion, is critical for reducing infant morbidity and mortality globally [28]. The literature suggests that there are substantial differences in vaccination between urban and rural areas [29, 30], with poorer vaccination coverage and timing in rural areas as compared to urban areas. In Armenia, however, the reverse was found. This could be ascribed to increased rural-to-urban migration which has been described in low- and middle-income countries with large numbers of impoverished families living in slums [31]. Data from two surveys, one conducted in the year 2000 and another in 2005 in Armenia, indicate that there has been a recent increase in internal migration in the country [32]. In the capital of Armenia (Yerevan), in the year 2000 21 % of the residents hailed from the countryside, whereas 35 % did in 2005 [32]. This has primarily been due to economic reasons [32]. Additionally, an increase in the proportion of women migrating to Yerevan has been reported with an increase in the number of families with non-co-resident men. These individuals might have poor access to healthcare [33, 34]. This pattern of internal migration and associated poor access to healthcare are a likely explanation of the lower timing of vaccination in urban Armenia, observed in our analysis. Similarly, in Kyrgyzstan, internal migration particularly to the capital city Bishkek, resulting in an increasing proportion of the urban poor, might account for the lower timing of vaccination in Bishkek versus other regions [35].

Mothers can be instrumental in gaining access to vaccination services for their children [36]. Previous studies have shown that maternal socio-demographic factors such as age and educational status are associated with correctly timed vaccinations [36, 37]. Improved vaccination rates have been reported with higher maternal education levels [30], which is in line with our findings. In our study, the likelihood of timely vaccination was higher among children with younger mothers. This concurs with the findings of Luman et al. [4, 36]. A possible explanation might be a higher awareness of vaccination among young mothers, since vaccination awareness has been found to be associated with vaccination coverage and completeness [38]. Additionally, older mothers are more likely to have more children, and caring for multiple children can in itself be a barrier to vaccination [36]. Luman et al. reported that mothers with two or three children were 20 % less likely and those with four or more children were 40 % less likely to have vaccinated children than those with only one child [36]. Other studies, however, report that the likelihood of properly timed vaccination increases with maternal age, which has been attributed to experience accumulated over time on the importance of vaccination and also on fatalities that might have occurred to children who were not vaccinated [37, 39, 40].

We found a higher vaccination coverage reported for measles, as per basic health statistics for the Member States of the WHO European Region [24], as compared to our estimates for both countries. In this case, the higher coverage might be partly attributable to the fact that children with contraindications to immunizations are excluded from official estimates of vaccination coverage in these countries [18]. This underscores the importance of having independent, additional sources of data such as the DHS to assess vaccination coverage and other health parameters.

### Limitations and strength of the study

Our findings are based on DHS survey data. Hence, our results depend on the quality of DHS data. DHS is the largest program for the collection of quantitative data on population and health from households in low- and middle-income countries and is considered to be one of the best sources of population-based information on health and health service utilization [40]. As far as the representativeness of the data is concerned, consistent sampling methods and questionnaires are used in DHS surveys in every country. We did not compare the two countries since the survey years and the observation periods varied, resulting in country-specific results that were not strictly comparable. We limited our analysis to vaccines for which data were available to assess changes in correctly timed vaccination. We were unable to assess timing for all nationally recommended vaccines.

Vaccination data in our analysis were obtained mostly from child health cards available at local health care facilities and by information recalled by the mother in the event that the mother did not have a child health card or an immunization was not recorded on the card. Hence, the possibility of recall bias and incompleteness of data bears mention. We applied the 1 minus the Kaplan-Meier function to estimate the proportion vaccinated by age, which is consistent with previous studies [3, 9, 29, 37]. This method enables visualizing vaccination uptake over time (or age) and provides estimates of the proportion vaccinated at a given age. This in turn makes it possible to monitor vaccination program effectiveness in terms of achieving target coverage rates [42]. However, this method consistently gives higher results than conventional methods due to censoring, as this method reduces the population at risk at the time point when censoring occurs. As the number of individuals under observation decreases with time, the right part of the curve becomes unstable and, accordingly, warrants careful interpretation [43].

## Conclusions

Our analysis suggests that vaccination coverage and correct timing in Armenia and Kyrgyzstan have improved over time. As a consequence of these developments, a reduction in vaccine-preventable diseases in both countries has likely occurred. As we near the approaching 2015 Millennium Development Goals deadline, these results are promising. However, socio-demographic and regional inequalities in vaccination timing persist.

## Abbreviations

DHS: Demographic and Health Survey
DTP: Diphtheria, tetanus, pertussis
MCV: Measles-containing vaccine
NIS: Newly Independent States
UNICEF: United Nations Children’s Fund
UTD vaccination coverage: Up-to-date vaccination coverage
WHO: World Health Organization.
